# Robust constrained weighted least squares for in vivo human cardiac diffusion kurtosis imaging

**DOI:** 10.1002/mrm.70037

**Published:** 2025-08-24

**Authors:** Sam Coveney, Maryam Afzali, Lars Mueller, Irvin Teh, Filip Szczepankiewicz, Derek K. Jones, Jürgen E. Schneider

**Affiliations:** ^1^ Biomedical Imaging Science Department, Leeds Institute of Cardiovascular and Metabolic Medicine University of Leeds Leeds UK; ^2^ Cardiff University Brain Research Imaging Centre (CUBRIC), School of Psychology Cardiff University Cardiff UK; ^3^ Medical Radiation Physics, Clinical Sciences Lund Lund University Lund Sweden

**Keywords:** cardiac diffusion tensor imaging, constrained estimation, diffusion kurtosis imaging, magnetic resonance imaging, robust estimation

## Abstract

**Purpose:**

Cardiac diffusion tensor imaging (cDTI) can investigate the microstructure of heart tissue. At sufficiently high b‐values, additional information on microstructure can be observed, but the data require a representation such as diffusion kurtosis imaging (DKI). cDTI is prone to image corruption, which is usually treated with shot rejection but which can be handled more generally with robust estimation. Unconstrained fitting allows DKI parameters to violate necessary constraints on signal behavior, causing errors in diffusion and kurtosis measures.

**Methods:**

We developed robust constrained weighted least squares (RCWLS) specifically for DKI. Using in vivo cardiac DKI data from 11 healthy volunteers collected with a Connectom scanner up to b‐value $1350\;s/mm^{2}$, we compared fitting techniques with/without robustness and with/without constraints.

**Results:**

Constraints, but not robustness, made a significant difference on all measures. Robust fitting corrected large errors for some subjects. RCWLS was the only technique that showed radial kurtosis to be larger than axial kurtosis for all subjects, which is expected in myocardium due to increased restrictions to diffusion perpendicular to the primary myocyte direction. For $b=1350\;s/mm^{2}$, RCWLS gave the following measures across subjects: mean diffusivity (MD) $1.68\pm 0.050\;\times 10^{-3}{\text{mm}}^{2}/s$, fractional anisotropy (FA) 0.30±0.013, mean kurtosis (MK) 0.36±0.027, axial kurtosis (AK) 0.26±0.027, radial kurtosis (RK) 0.42±0.040, and RK/AK 1.65±0.19.

**Conclusion:**

Fitting techniques utilizing both robust estimation and convexity constraints, such as RCWLS, are essential to obtain robust and feasible diffusion and kurtosis measures from in vivo cardiac DKI.

## 1 INTRODUCTION

Cardiac diffusion weighted imaging (cDWI) is a magnetic resonance imaging (MRI) technique that can be used to investigate cardiac tissue microstructure. Cardiac diffusion tensor imaging (cDTI) is the most common cDWI method used on the heart, from which measures such as mean diffusivity (MD) and fractional anisotropy (FA) can be derived. However, the diffusion‐weighted signal in tissue deviates from monoexponential decay at higher diffusion weighting (as expressed by the b‐values) due to cell membranes and other restrictions in biological tissue.^1^, ^2^, ^3^ Diffusion kurtosis imaging (DKI) can quantify these deviations. Non‐Gaussian diffusion models (including DKI) have been shown to have a higher sensitivity for the detection of hypertrophy in ex vivo rat hearts compared with DTI.^3^ Kurtosis measures include mean kurtosis (average kurtosis across all directions), axial kurtosis (kurtosis in the primary diffusion direction) and radial kurtosis (average kurtosis in the plane perpendicular to axial kurtosis).^4^, ^5^, ^6^, ^7^, ^8^, ^9^, ^10^, ^11^, ^12^ Anisotropic and isotropic kurtosis can be also distinguished with q‐space trajectory imaging.^13^

Spin echo based DKI in the human heart in vivo is challenging due to low SNR, a short myocardial T2 (approximately 46 ms at 3.0 T^14^) and long echo‐times (TE), required to achieve sufficient motion compensation and b‐values. Nonetheless, acquiring data with sufficiently high b‐values for cardiac DKI has been shown to be feasible in healthy volunteers in vivo using ultra‐strong gradients (i.e. 300 mT/m) at echo times and resolutions comparable to those commonly used for conventional cDTI.^15^, ^16^, ^17^ However, even for the brain, which has longer T2 and significantly less motion, DKI is challenging: fitting methods need to handle data corruptions,^18^ but also need to yield a physically plausible signal if kurtosis measures are to be meaningful.^19^

Image corruption is a common problem in cDWI.^20^ Furthermore, motion causes additional signal variations sometimes referred to as physiological noise. In cDTI, shot rejection is usually performed in an attempt to handle corruptions where noticeably corrupted images are removed from datasets before fitting, a method that is typically time consuming and subjective. In our experience, the reduced signal at higher b‐values simultaneously causes a larger number of corruptions (including those from misregistration) and a decreased ability to perform shot‐rejection effectively. Robust estimation, in which outlier signals are identified and removed at the voxel level, is an alternative to shot rejection. Our recent work shows that robust estimation is superior to shot rejection in cDTI,^21^ so robust estimation in cardiac DKI is worth investigating, but has not been done yet.

Although the DKI signal representation does not correspond to a valid diffusion propagator, the fitted signal should still adhere to the physical principles governing the data‐generating process. If it does not, the fitted parameters and any measures derived from them will lack meaningful interpretation. For example, the compartment model predicts that kurtosis should be nonnegative,^2^, ^4^ and the diffusion tensor should be positive definite as in DTI. For DKI, it is not known how to enforce constraints on kurtosis via reparameterization of the fitting problem, so constraints must consider whether the predicted signal behavior is valid, for example, in,^22^ nonlinear optimization is (infinitely) penalized if constraints are violated. Recently, linear least squares methods have been developed for enforcing convexity constraints on the cumulant generating function, leading to correction of significant errors in brain DKI.^19^ These advantages should also apply to cardiac DKI.

While preventing estimated parameters from violating constraints may be seen as a form of robustness, constrained fitting itself is not inherently robust. This is because the constraints do not change the shape of the fitting cost function in parameter space, which is entirely determined by the data. As a result, outliers can still have a detrimental impact on parameter estimates. Furthermore, although robust fitting may reduce the frequency and impact of constraint violations by removing outlier signals, it cannot guarantee that the parameters that optimize the cost function will not violate the constraints. The objectives of this work are (1) to demonstrate a way of combining robust fitting^21^ with convexity constraints^19^ using iteratively reweighted least squares (IRLS) to give robust constrained weighted least squares (RCWLS) and (2) to test various fitting methods (with/without robustness and with/without constraints) on in vivo cardiac DKI data collected on a Connectom scanner to determine the effects on diffusion and kurtosis measures. To our knowledge, this is the first time that constrained estimation has been combined with robust estimation in MRI.

## 2 METHODS

We use the following notation: tensors are bold and uppercase; vectors are bold and lowercase; tensor and vector elements are italicized and indexed.

### 2.1 Diffusion kurtosis imaging

The DKI signal representation can be expressed as^2^:

(1) $$\begin{array}{ll} \ln S(q) & =\ln S_{0}-\overset{3}{\underset{i=1}{\sum }}\overset{3}{\underset{j=1}{\sum }}q_{i}q_{j}D_{ij}\\ & \;+\frac{1}{6}{\bar{D}}^{2}\overset{3}{\underset{i=1}{\sum }}\overset{3}{\underset{j=1}{\sum }}\overset{3}{\underset{k=1}{\sum }}\overset{3}{\underset{l=1}{\sum }}q_{i}q_{j}q_{k}q_{l}W_{ijkl} \end{array}$$

where $q=\sqrt{b}\cdot \left(n_{1},n_{2},n_{3}\right)$ is the (rescaled) wave vector (denoted this way for convenience in expressing the constraints—see Section 2.3), and i,j,k,l index physical space coordinates. The diffusion tensor D and kurtosis tensor W are both symmetric, having 6 and 15 unique elements, respectively. The signal at $b=0s/mm^{2}$ is denoted by scalar quantity $S_{0}$. The DTI signal representation is the same as Equation (1) but without the term containing W. Kurtosis is expressed in dimensionless form due to scaling by the mean diffusivity $\bar{D}=\left(D_{11}+D_{22}+D_{33}\right)/\;3$.

Expanding Equation (1) accounting for the symmetry of D and W gives the following linear expression:

(2a) $$\begin{array}{ll} f_{\theta }(q)\equiv \ln S(q) & =\theta^{T}\cdot \;x \end{array}$$

(2b) $$\begin{array}{ll} \theta & ={\left(\theta_{1},\theta_{2},\ldots ,\theta_{22}\right)}^{T} \end{array}$$

(2c) $$\begin{array}{ll} x & =\left(-q_{1}^{2},-2q_{1}q_{2},-q_{2}^{2},-2q_{1}q_{3},-2q_{2}q_{3},-q_{3}^{2}\right.,\\ & \;\frac{1}{6}q_{1}^{4},\frac{1}{6}q_{2}^{4},\frac{1}{6}q_{3}^{4},\frac{4}{6}q_{1}^{3}q_{2},\frac{4}{6}q_{1}^{3}q_{3},\frac{4}{6}q_{2}^{3}q_{1},\\ & \;\frac{4}{6}q_{2}^{3}q_{3},\frac{4}{6}q_{3}^{3}q_{1},\frac{4}{6}q_{3}^{3}q_{2},q_{1}^{2}q_{2}^{2},q_{1}^{2}q_{3}^{2},q_{2}^{2}q_{3}^{2},\\ & \;2q_{1}^{2}q_{2}q_{3},{\left.2q_{2}^{2}q_{1}q_{3},2q_{3}^{2}q_{1}q_{2},-1\right)}^{T} \end{array}$$

The 22 coefficients in θ are related to the original parameters as follows: 6 diffusion tensor parameters $\left(\theta_{1},\ldots ,\theta_{6}\right)=\left(D_{11},D_{12},D_{22},D_{13},D_{23},D_{33}\right)$, 15 kurtosis tensor parameters $\left(\theta_{7},\ldots ,\theta_{21})={\bar{D}}^{2}\cdot (W_{1111},W_{2222},W_{3333},W_{1112},W_{1113},W_{1222},W_{2223},W_{1333},W_{2333},W_{1122},W_{1133},W_{2233},W_{1123},W_{1223},W_{1233}\right)$, and intercept $\theta_{22}=-\ln S_{0}$. The DTI expression would include only terms depending on $\left(\theta_{1},\ldots ,\theta_{6}\right)$ and $\theta_{22}$.

### 2.2 Weighted least squares

Given N observations $\left\{q_{n},S_{n}|n=1\ldots N\right\}$, the weighted least squares (WLS) estimate of the coefficients θ in Equation (2), denoted by ${\hat{\theta }}_{\text{WLS}}$, is given by:

(3) $$\begin{array}{ll} {\hat{\theta }}_{\text{WLS}} & ={arg\;min}_{\theta }\overset{N}{\underset{n=1}{\sum }}w_{n}{\left(f_{\theta }(q_{n})-\ln S_{n}\right)}^{2} \end{array}$$

The wavevectors $q_{n}$ of the nth observation can be converted to $x_{n}$ using Equation (2c). Given design matrix $X={\left(x_{1},x_{2},\ldots ,x_{N}\right)}^{T}$, observation vector $y=(\ln S_{1},\ln S_{2},\ldots ,{\ln S_{N})}^{T}$, and weights vector $w={\left(w_{1},w_{2},\ldots ,w_{N}\right)}^{T}$, a weighted design matrix and observation vector can be defined:

(4) $$X^{\prime }=\text{diag}\left(\sqrt{w}\right)\cdot X\;,\;y^{\prime }=\text{diag}\left(\sqrt{w}\right)\cdot y$$

The WLS estimate can then be written as

(5) $$\begin{array}{ll} {\hat{\theta }}_{\text{WLS}} & ={arg\;min}_{\theta }||X^{\prime }\cdot \theta -y^{\prime }|{|}^{2}\\ & ={\left({X^{\prime }}^{T}\cdot X^{\prime }\right)}^{-1}\cdot X^{\prime }\cdot y^{\prime } \end{array}$$

For uniform weights $w_{n}=1$, Equation (5) gives the ordinary least squares (OLS) estimate ${\hat{\theta }}_{\text{OLS}}$.

### 2.3 Convexity constraints

A useful constraint for DKI is to enforce convexity of the cumulant generating function 𝒞(q) ^19^:

(6) $$𝒞(q):=\ln S(\sqrt{-1}q)\equiv f_{\theta }(\sqrt{-1}q)$$

This constraint can be enforced by using sum of squares polynomials^19^; the mathematical background for this can be found in.^23^ The semi‐definite program for solving the WLS problem subject to constraints, thus yielding the constrained WLS estimate ${\hat{\theta }}_{\text{CWLS}}$, can be written as follows:

(7a) $$\begin{array}{ll} {\hat{\theta }}_{\text{CWLS}} & ={arg\;min}_{\theta ,\alpha }||X^{\prime }\cdot \theta -y^{\prime }|{|}^{2} \end{array}$$

(7b) $$\begin{array}{ll} \text{subject to}\; & e^{T}\cdot \left(H(\theta )+L(\alpha )\right)\cdot e=h_{\theta }(q,s) \end{array}$$

(7c) $$\begin{array}{ll} & H(\theta )+L(\alpha )≽0 \end{array}$$

(7d) $$\begin{array}{ll} \text{where}\; & h_{\theta }(q,s):=s^{T}\cdot H_{𝒞}(q)\cdot s \end{array}$$

(7e) $$\begin{array}{ll} & e^{T}\cdot H(\theta )\cdot e=h_{\theta }(q,s) \end{array}$$

(7f) $$\begin{array}{ll} & e^{T}\cdot L(\alpha )\cdot e=0 \end{array}$$

Importantly, we have written the problem in terms of the weighted design matrix $X^{\prime }$ and observation vector $y^{\prime }$.

We will briefly explain Equation (7) for DKI, leaving details on the constraint matrices H(θ) and L(α) (presented here for the first time) for Appendix B. Equation (7d) defines $h_{\theta }(q,s)$, where $H_{𝒞}(q)$ is the Jacobian of 𝒞(q) and the dummy variable s has the same dimensions as q. Then, $h_{\theta }(q,s)$ is just a polynomial. Convexity of 𝒞(q) requires that $h_{\theta }(q,s)$ is nonnegative, which can be enforced using a sum of squares polynomial representation, that is, Equation (7b) (see^23^). We can *exactly* represent $h_{\theta }(q,s)$ using a relatively small monomial basis for e (see Appendix B). The convexity constraint is satisfied when Equation (7c) holds, that is, when H(θ)+L(α) is positive semi‐definite (PSD). Note that Equation (7) involves optimizing over coefficients θ and slack parameters α. Appendix C explains how the numerical complexity of the problem can be reduced.

### 2.4 Robust fitting

In DTI/DKI, WLS usually refers to solving Equation (5) by weighting the (squared) residuals of the linearized problem with the (squared) signal^24^:

(8) $$w_{n}={\left(\exp f_{\theta }(q_{n})\right)}^{2}\leftarrow \;{\hat{\theta }}_{\text{OLS}}$$

where an initial OLS estimate ${\hat{\theta }}_{\text{OLS}}$ is used to predict the signals (since the true signals are not known). Henceforth, we will use “WLS” to refer to Equation (5) using the weights in Equation (8). Correspondingly, “CWLS” will refer to constrained WLS, that is, solving Equation (7) using the weights given by Equation (8).

Importantly, neither WLS nor CWLS are intrinsically robust, and outlier data can have a detrimental effect on the estimates ${\hat{\theta }}_{\text{WLS}}$ or ${\hat{\theta }}_{\text{CWLS}}$. Robust estimation can be implemented using iteratively reweighted least squares (IRLS), using weights derived from a robust estimator.^18^, ^21^, ^25^ Notably, for WLS in DTI/DKI, these robust weight should be chosen so as to preserve the cost function implied by Equations (3) and (8) (see^21^ for a derivation), such that IRLS solves the WLS/CWLS problem in a robust way. Robust fitting in the DTI/DKI literature is usually done in order to remove the influence of outlier data on the fitted signal, thus making it easier to identify outlier data so that the original problem can be solved without robust weights but also without the outliers.

A robust weighting scheme accounting for the DTI/DKI weights in Equation (8), based on the Geman–McClure M‐estimator, with K iterations, is^21^, ^25^:

(9a) $$\begin{array}{ll} w_{n}^{1} & ={\left(\exp f_{\theta }(q_{n})\right)}^{2}\leftarrow \;{\hat{\theta }}_{\text{OLS}} \end{array}$$

(9b) $$\begin{array}{ll} w_{n}^{k} & ={\left(\frac{\hat{\sigma }/\exp f_{\theta }(q_{n})}{{\left(\hat{\sigma }/\exp f_{\theta }(q_{n})\right)}^{2}+u_{n}^{2}}\right)}^{2}\leftarrow {\left(\begin{array}{c} {\hat{\theta }}_{*}\\ u_{n}\\ \hat{\sigma } \end{array}\right)}^{k-1} \end{array}$$

(9c) $$\begin{array}{ll} w_{n}^{K-1} & =\left\{\begin{array}{c} 1\;\text{if}\;y_{n}\notin O\;\\ 0\;\text{if}\;y_{n}\in O\; \end{array}\right. \end{array}$$

(9d) $$\begin{array}{ll} w_{n}^{K} & =\left\{\begin{array}{l} \begin{array}{ccc} & {\left(\exp f_{\theta }(q_{n})\right)}^{2}\leftarrow \;{\hat{\theta }}_{*}^{K-1} & \text{if}\;y_{n}\notin 𝒪\\ & 0 & \text{if}\;y_{n}\in 𝒪 \end{array} \end{array}\right. \end{array}$$

where ${\hat{\theta }}_{*}^{k}$ corresponds to the estimated coefficients for iteration k (we define $u_{n}^{k}$ and ${\hat{\sigma }}^{k}$ below). This scheme requires at least 4 iterations (in which case, a single robustly weighted fit will have been performed for k=2). As with Equation (8), the symbol ← in Equation (9) is used to mean that the quantities on the right are used to evaluate the expression for the weights on the left, for example, $w_{n}^{2}$ would be calculated using ${\hat{\theta }}_{*}$, $u_{n}$, and $\hat{\sigma }$ from the first iteration. The residuals of the WLS problem are defined as the difference between the log observed signal and log predicted signal:

(10) $$u_{n}^{k}:=y_{n}-f_{\theta }(q_{n})\leftarrow {\hat{\theta }}_{*}^{k}$$

The noise level $\hat{\sigma }$ is estimated at the kth iteration using a robust estimator designed for the WLS problem^25^:

(11) $$\begin{array}{l} {\hat{\sigma }}^{k}=\frac{1.4826\;N}{N-m}\times \text{MED}\left[|z_{n}-\text{MED}\left[z_{n}]|\right.\right] \end{array}$$

where $z_{n}\equiv \exp(f_{\theta }(q_{n}))u_{n}\leftarrow {\hat{\theta }}_{*}^{k}$, MED is the median operator and m is the number of regressors, that is, m=7 for DTI and m=22 for DKI.

Set 𝒪 contains log‐signals defined as outliers by a 3‐sigma rule, applied after the last robustly‐weighted fit:

(12) $$\begin{array}{l} y_{n}\in 𝒪\;\text{if}\;|\exp(y_{n})-\exp(f_{\theta }(q_{n}))|>3{\hat{\sigma }}^{K-2} \end{array}$$

such that the estimated coefficients ${\hat{\theta }}_{*}$ at iteration K−2 are used to evaluate $f_{\theta }(q_{n})$. If the nth log‐signal $y_{n}$ is defined as an outlier, then it receives a weight of zero in the last two iterations in Equation (9).

The main insight in this paper is that we can use IRLS with a robust weighting scheme designed specifically for DTI/DKI, that is, Equation (9), but we are free to choose whether to estimate the coefficients at each iteration using (unconstrained) WLS with Equation (5) or CWLS with Eqs (7). The constraints are independent of the weights, which only enter the cost function Equation (7a) through Equation (4). We will refer to IRLS with weights given by Equation (9) as robust WLS (RWLS) if Equation (5) is used at each iteration, or as robust constrained WLS (RCWLS) if Equation (7) is used at each iteration. The estimated coefficients obtained from the last iteration with weights $w^{K}$ are denoted as ${\hat{\theta }}_{\text{RWLS}}$ for RWLS and ${\hat{\theta }}_{\text{RCWLS}}$ for RCWLS. For convenience, the unconstrained OLS estimate ${\hat{\theta }}_{\text{OLS}}$ is used to define weights for the first iteration for both RWLS and RCWLS. We modified DiPy^26^ to be able to solve RWLS and RCWLS. These modifications have been incorporated in DiPy as of v1.10.

### 2.5 Experimental setup and recruitment

Cardiac diffusion‐weighted images (cDWI) were acquired on a Connectom 3T research‐only MR imaging system (Siemens Healthcare, Erlangen, Germany) with a maximum gradient strength of 300 mT/m and slew rate of 200 T/m/s. An 18‐channel body receive coil was used in combination with a 32‐channel spine receive coil. Eleven healthy volunteers (with no known previous cardiac conditions) were recruited for this study: age range 20.5±1.9 years (18−24 years), weight range 64.1±11.4 kg (54−94 kg), 7 females. The study was approved by the local institutional review board (Cardiff University School of Psychology Research Ethics Committee) and all subjects provided written consent.

**FIGURE A1:**
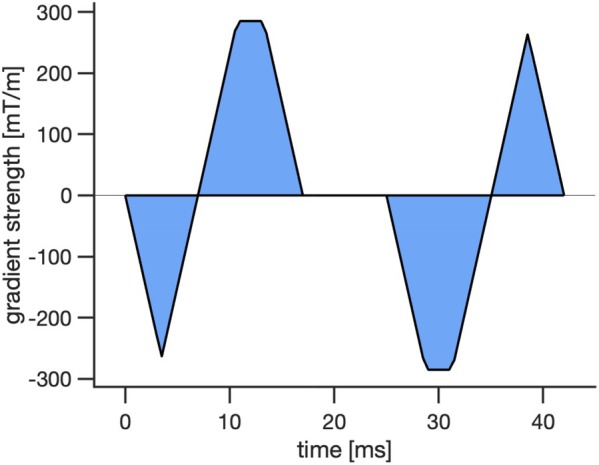
Numerically optimized second‐order motion compensated waveform for $b_{\max}=1350\;s/mm^{2}$, $G_{\max}=285.4\;\mathrm{mT}/m$ and maximum slew rate 76.2T/m/s.

A prototype pulse sequence was used that enables diffusion encoding with user‐defined second‐order motion‐compensated (M0M1M2) diffusion gradient waveforms, designed with the NOW toolbox^27^, ^28^, ^29^, ^30^ (see Figure A1). The maximum gradient strength used in this study for the M0M1M2 waveform to generate the b‐value of $1350\;s/mm^{2}$ was 285.4 mT/m, and the maximum physiologically limited slew rate was 76.2 T/m/s.^15^ The cDWI parameters were TR = 3 RR‐intervals, TE = 61ms, EPI readout, field‐of‐view = $320\times 120\;mm^{2}$ using ZOnally‐magnified Oblique Multislice (ZOOM, tilted RF: excitation, tilt angle: 15°, tilted slice thickness: 20mm),^29^, ^31^ in‐plane resolution = $2.7\times 2.7\;mm^{2}$, slice thickness = 8mm, 3 short axis slices (base, mid, and apical), partial Fourier factor = 7/8, no parallel imaging, bandwidth = 2354 Hz/pixel. Each full dataset comprised of 5 b‐values (b = 100, 450, 900, 1200, 1350 $s/mm^{2}$). For $b\geq 450\;s/mm^{2}$, 30 directions per shell were acquired with 6 repetitions while $b=100\;s/mm^{2}$ consisted of 3 directions with 12 repeats. Data were acquired with ECG‐gating and under free‐breathing (respiratory navigators were not employed for this work). The trigger delay was adjusted for cDWI acquisition in mid‐end systole. Saturation bands were placed around the heart. Fat suppression was performed using the SPAIR method.^32^ The scan time for all the diffusion weighted images was around 40 min depending on subject heart rate. Including cardiac planning, the total scan time was around one hour.

### 2.6 Post‐processing

Post‐processing was done using in‐house tools,^20^ with rigid image registration utilizing SITK^33^ and fitting utilizing DiPy (with our updates).^26^ Image registration was performed by masking a suitable $b=100\;s/mm^{2}$ image, registering all $b=100\;s/mm^{2}$ images to this reference image, then using the average of registered $b=100\;s/mm^{2}$ images to register the entire dataset. Correlation was used as the registration metric, since it outperformed mutual information for high b‐value images. The DTI signal representation was then fit to $b\leq 450\;s/mm^{2}$ images using RWLS, and the full image series was predicted. Each original (unmodified) image was then registered to the corresponding predicted image. This method, similar to,^34^ improved the registration.

After registration, we fit the DTI signal representation to the $b\leq 450\;s/mm^{2}$ data using RWLS. MD, FA, and helix angle (HA) (using a cylindrical coordinate system with origin on the LV blood‐pool center) were calculated. Segmentation of the LV contours was performed with care taken to exclude voxels exhibiting strong partial‐volume effects. For regions strongly affected by artifacts, such as aliasing or susceptibility‐induced warping, fitting results do not reliably represent tissue properties. Artifact masks were defined using sectors centered on the LV blood pool, in order to ignore these parts of the myocardium when calculating voxel statistics. This masking was performed by considering the image series, as well as utilizing MD, FA, HA, root mean square error and coefficient of determination (R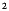) from the RWLS DTI fit to $b\leq 450\;s/mm^{2}$ data. Across all subjects an average of 25% of voxels were excluded. We have not utilized DKI results to identify artifacts in any way since this would likely bias comparison between the fitting methods under study.

Having registered the images and segmented the myocardium, we performed further fitting experiments on myocardial voxels only. Defining $b_{\max}$ as the maximum b‐value images that were utilized in a given fit (such that all images with a lower b‐value were also included), we performed the following: DTI using WLS and RWLS for $b_{\max}=450\;s/mm^{2}$; DKI with WLS, RWLS, CWLS, and RCWLS, for $b_{\max}$ values 900, 1200, and $1350\;s/mm^{2}$. For RWLS and RCWLS, we used K=10 iterations. We calculated the following measures in each voxel: mean diffusivity (MD), fractional anisotropy (FA), mean kurtosis (MK), axial kurtosis (AK), radial kurtosis (RK), and radial / axial kurtosis (RK/AK).^2^, ^4^ We then calculated the average of these measures over non‐artifact myocardial voxels.

### 2.7 Statistical methods

In order to make tractable comparisons between different fitting methods and to isolate the specific effects of constraints and robustness, we applied paired tests to measures obtained from different fitting methods for the same $b_{\max}$, and different $b_{\max}$ for the same fitting method. Specifically, we used the Wilcoxon signed‐rank test since non‐robust methods often produced results that violate the assumption of normality (which was tested with the Shapiro‐Wilk test).

In this work, we potentially face the “multiple comparisons problem” since we compare many diffusion and kurtosis measures, for many $b_{\max}$ values and fitting methods. It is not obvious how to adjust significance levels in the context of comparing multiple fitting methods on the same data (particularly given that multiple different measures are calculated from the same set of estimated coefficients, and that higher $b_{\max}$ fits included all data with lower b‐values). Methods for adjustment of significance levels rely on independence assumptions that do not seem to apply here, especially for a comparison of fitting methods. We therefore take care to draw conclusions that are supported by the overall results.

## 3 RESULTS

### 3.1 Group analysis

**FIGURE 1:**
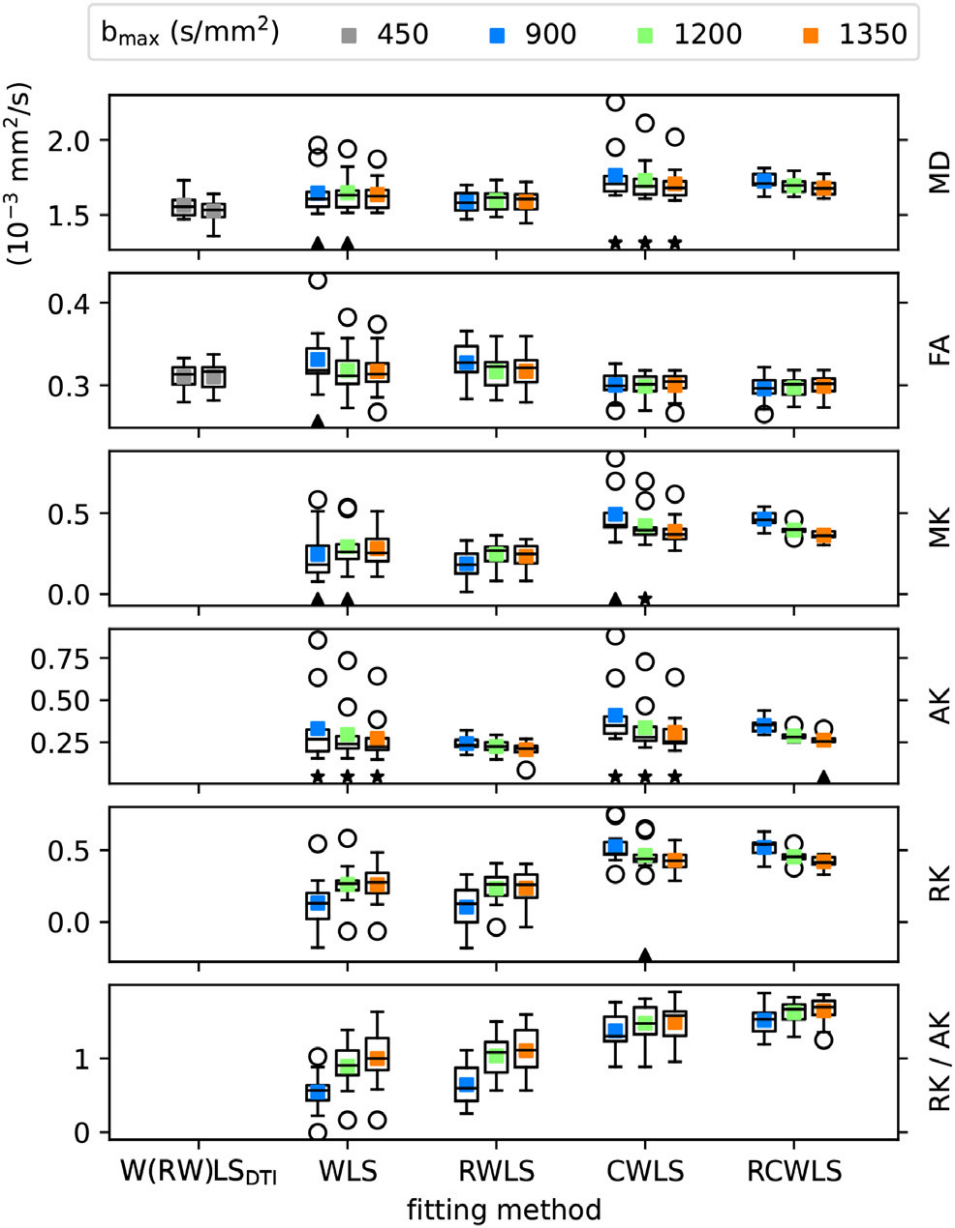
Average of DTI/DKI measures over myocardial voxels for all subjects. Colored points show the average over subjects, colored by $b_{\max}$. DTI fits ${\text{W(RW)LS}}_{\text{DTI}}$ show WLS (left) and RWLS (right) together for convenience. The black triangles (stars) show where the Shapiro‐Wilk test *p*‐value ≤0.05 (≤0.01), that is, the hypothesis of normality is rejected.

**FIGURE 2:**
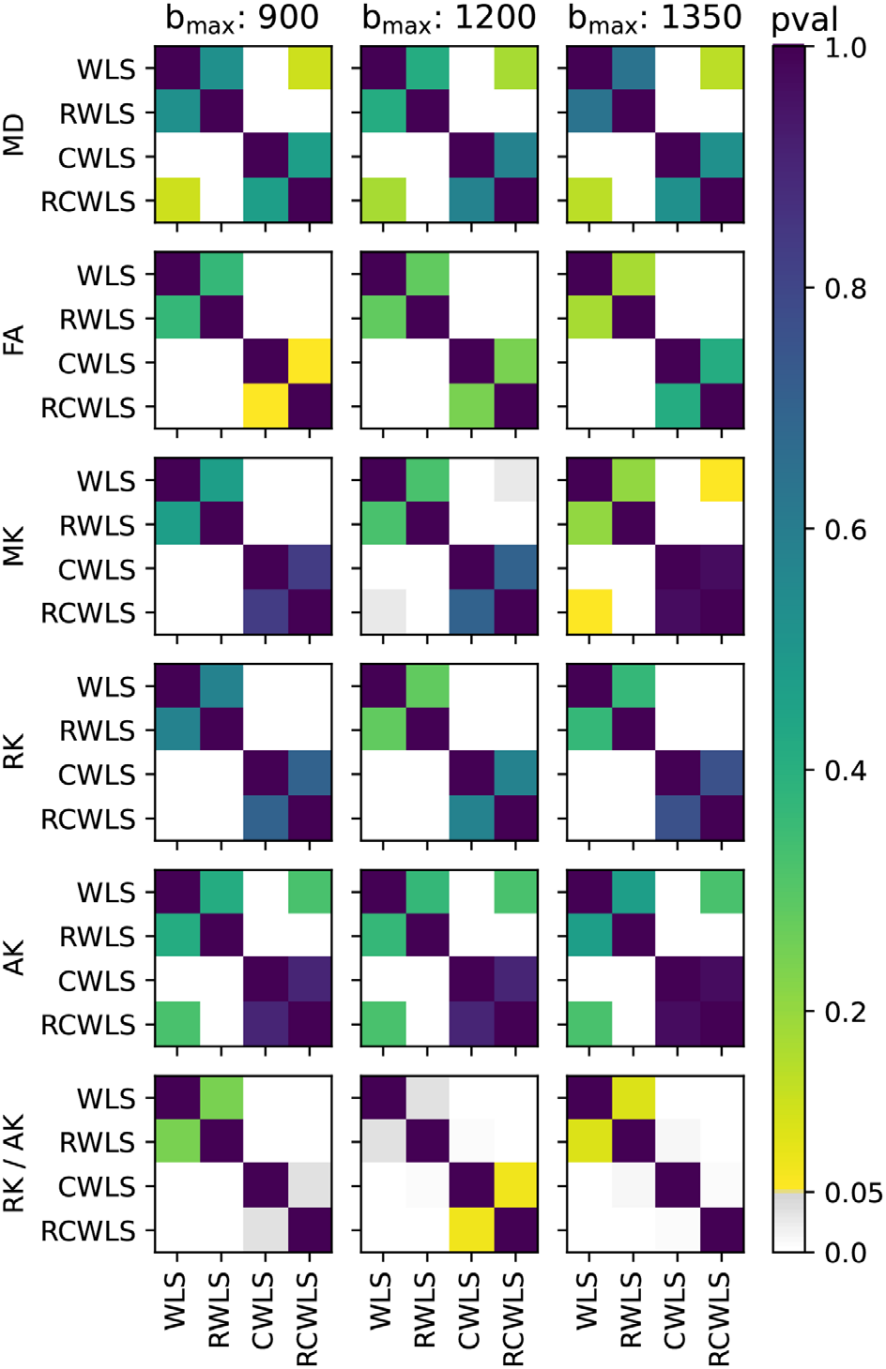
*p*‐values from comparing DKI measures from different fitting methods given the same $b_{\max}$ (units $s/mm^{2}$).

**FIGURE 3:**
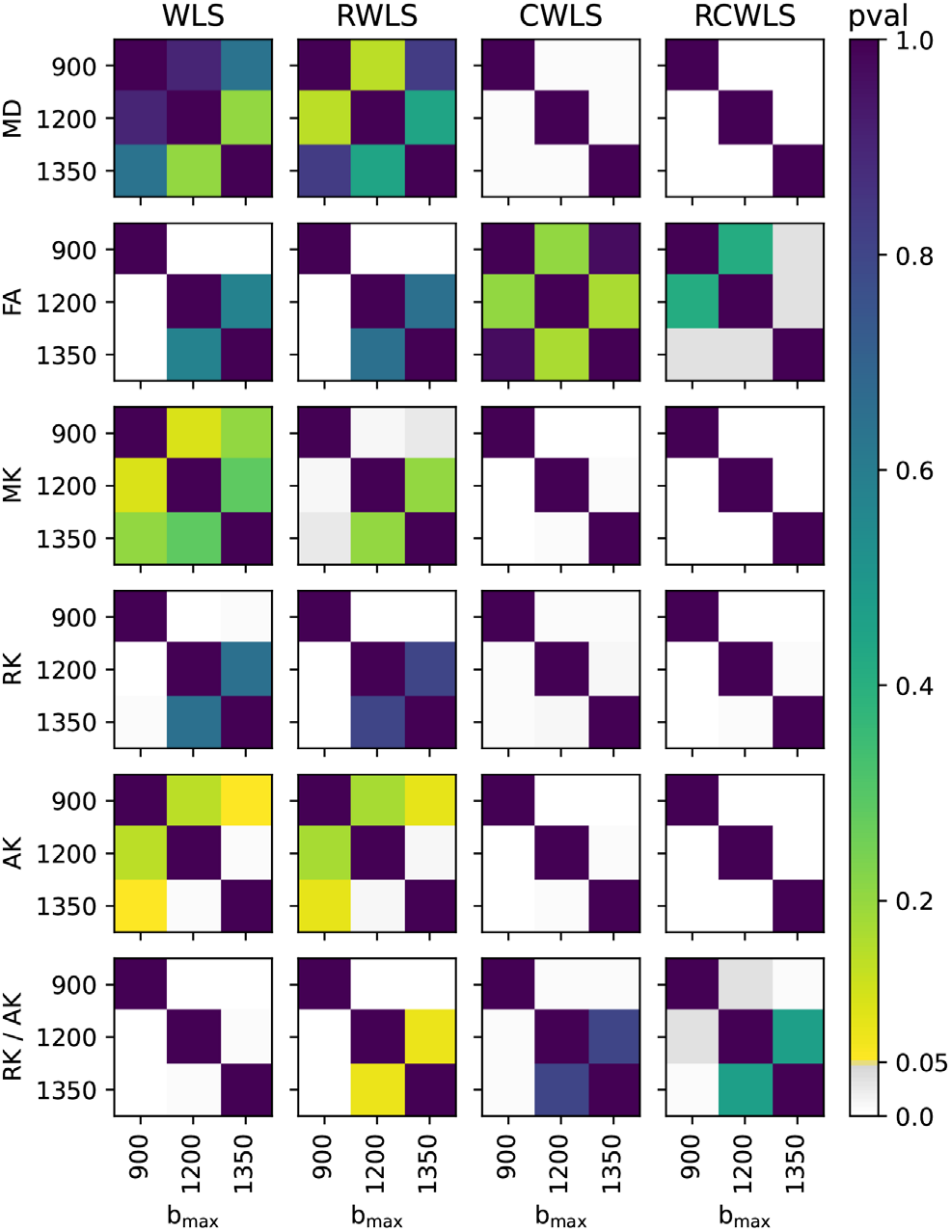
*p*‐values from comparing DKI measures from different $b_{\max}$ (units $s/mm^{2}$) given the same fitting methods.

**FIGURE 4:**
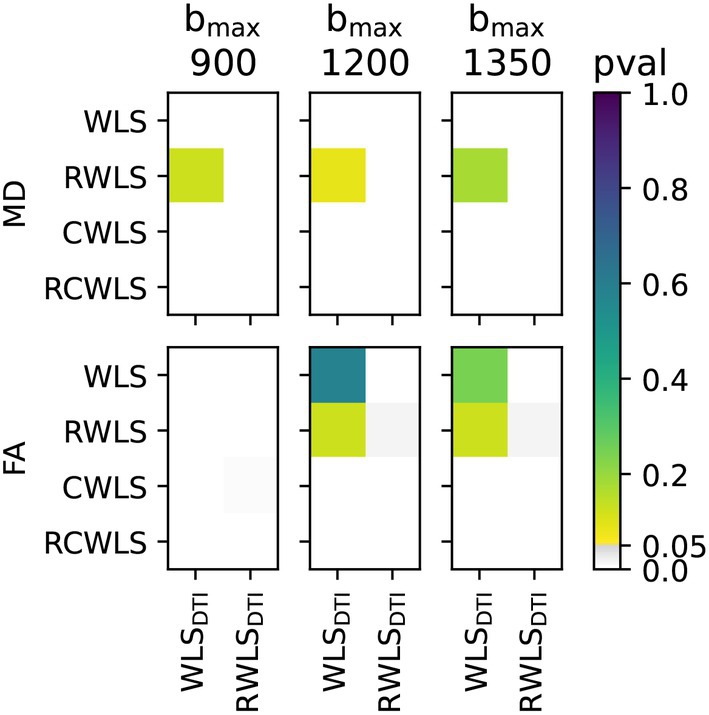
*p*‐values from comparing MD and FA from DKI fitting methods for each $b_{\max}$ used for DKI fitting (units $s/mm^{2}$) against DTI fitting methods ($b_{\max}=450\;s/mm^{2}$).

Figure 1 shows boxplots of the average DTI/DKI measures for all 11 subjects. All fitting methods are shown for all $b_{\max}$ values. The black markers drawn along the bottom of each subplot indicate non‐normality of the data in each boxplot. Figures 2, 3, 4 show the results of significance tests between measures from the different fitting methods and different $b_{\max}$ shown in Figure 1. Figure 2 shows the *p*‐values between different methods within each $b_{\max}$ in order to demonstrate whether different methods make a significant difference given the same $b_{\max}$. Figure 3 shows the *p*‐values between different $b_{\max}$ within each method, to see whether $b_{\max}$ made a significant difference in measures given the method. Additionally, Figure 4 shows *p*‐values between DKI methods and DTI methods for MD and FA only, grouped by $b_{\max}$ of the DKI fits. For $b=1350\;s/mm^{2}$, RCWLS gave the following measures across subjects: MD $1.68\pm 0.050\;\times 10^{-3}{\text{mm}}^{2}/s$, FA 0.30±0.013, MK 0.36±0.027, AK 0.26±0.027, RK 0.42±0.040, and RK/AK 1.65±0.19.

#### 3.1.1 Constraints

(i) Adding constraints to a method, that is, going from WLS to CWLS and from RWLS to RCWLS, made a statistically significant difference on all measures, for all $b_{\max}$ values, as shown in Figure 2. Figure 1 shows that constraints increased all kurtosis measures MK, AK, RK, and RK/AK, the latter results showing that constraints result in increasing RK more than AK. Increased kurtosis is consistent with the constraints ensuring that fitted parameters correspond to nonnegative kurtosis. (ii) For constrained fitting, MK, RK, and AK all decrease with $b_{\max}$, with Figure 3 showing these changes are significant for both CWLS and RCWLS. However, Figure 1 indicates that the ratio RK/AK appears to increase with $b_{\max}$ for both constrained and unconstrained fitting, although this difference is not significant between $b_{\max}=1200\;s/mm^{2}$ and $b_{\max}=1350\;s/mm^{2}$.

#### 3.1.2 Robustness

(i) Robust fitting by itself, that is, going from WLS to RWLS, and CWLS to RCWLS, gave large changes in measures for some subjects (in particular, reducing MD and MK) but did not significantly change the mean measure values over subjects. Robust fitting generally reduces the spread of the measures over the group by correcting errors in measures for some subjects, as is visually clear in Figure 1. Non‐normality of measures was only found for non‐robust methods (with the *single exception*, out of such 36 tests, being AK for RCWLS at $b_{\max}=1350\;s/mm^{2}$). (ii) Robustness *may* has an effect on RK/AK; Figure 2 shows the following *p*‐values comparing CWLS and RCWLS: $b_{\max}=900\;s/mm^{2}$ (p=0.032), $b_{\max}=1200\;s/mm^{2}$ (p=0.068), and $b_{\max}=1350\;s/mm^{2}$ (p=0.024). However, in the context of multiple comparisons, where no effect of robustness was seen on other measures and a 0.05 significance threshold was not met for all $b_{\max}$, this result cannot be ascribed significance.

#### 3.1.3 MD and FA

For RWLS DTI fits, we obtained MD $1.53\pm 0.074\;\times 10^{-3}{\text{mm}}^{2}/s$ and FA 0.31±0.017. When considering robust methods, (i) MD increases from DTI ($b_{\max}\leq 450\;s/mm^{2}$) to unconstrained DKI (see Figure 4), and from unconstrained DKI to constrained DKI (see Figure 2); FA increases from DTI to unconstrained DKI (see Figure 4) but constrained DKI results in lower FA than DTI fitting (see Figure 4). (ii) For constrained methods, as $b_{\max}$ increases MD decreases slightly, but there is little change in FA (see Figure 3).

### 3.2 Example maps

**FIGURE 5:**
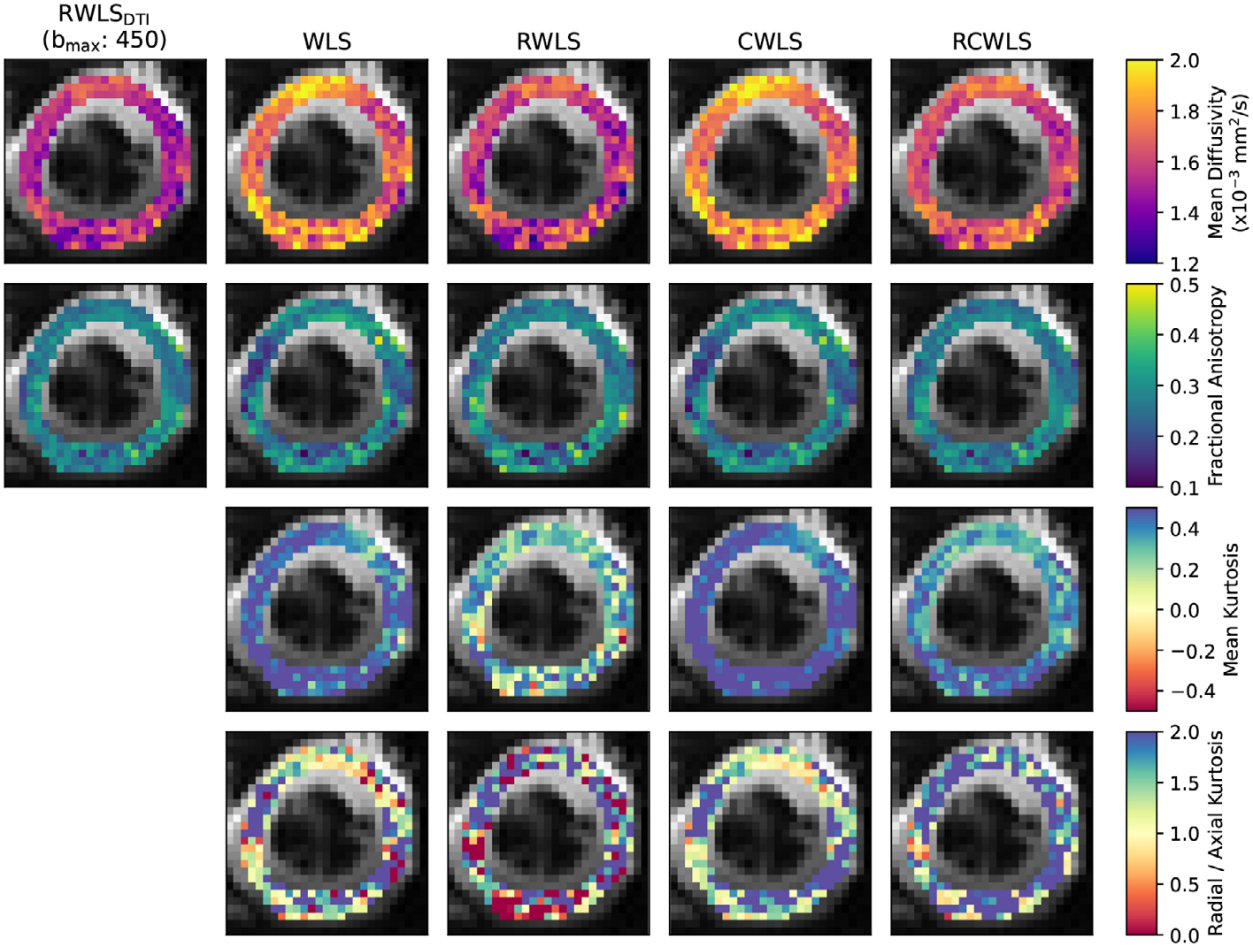
Maps for the mid slice of a single subject, for $b_{\max}=1350\;s/mm^{2}$. The first column shows MD and FA from DTI RWLS fitting ($b_{\max}=450\;s/mm^{2}$) for reference. All other columns show measures from DKI fits. In this example, robust fitting decreases MK, while constrained fitting increases MK. Using robust and constrained fitting (RCWLS) gives the most plausible results, with mostly RK/AK > 1 values.

**FIGURE 6:**
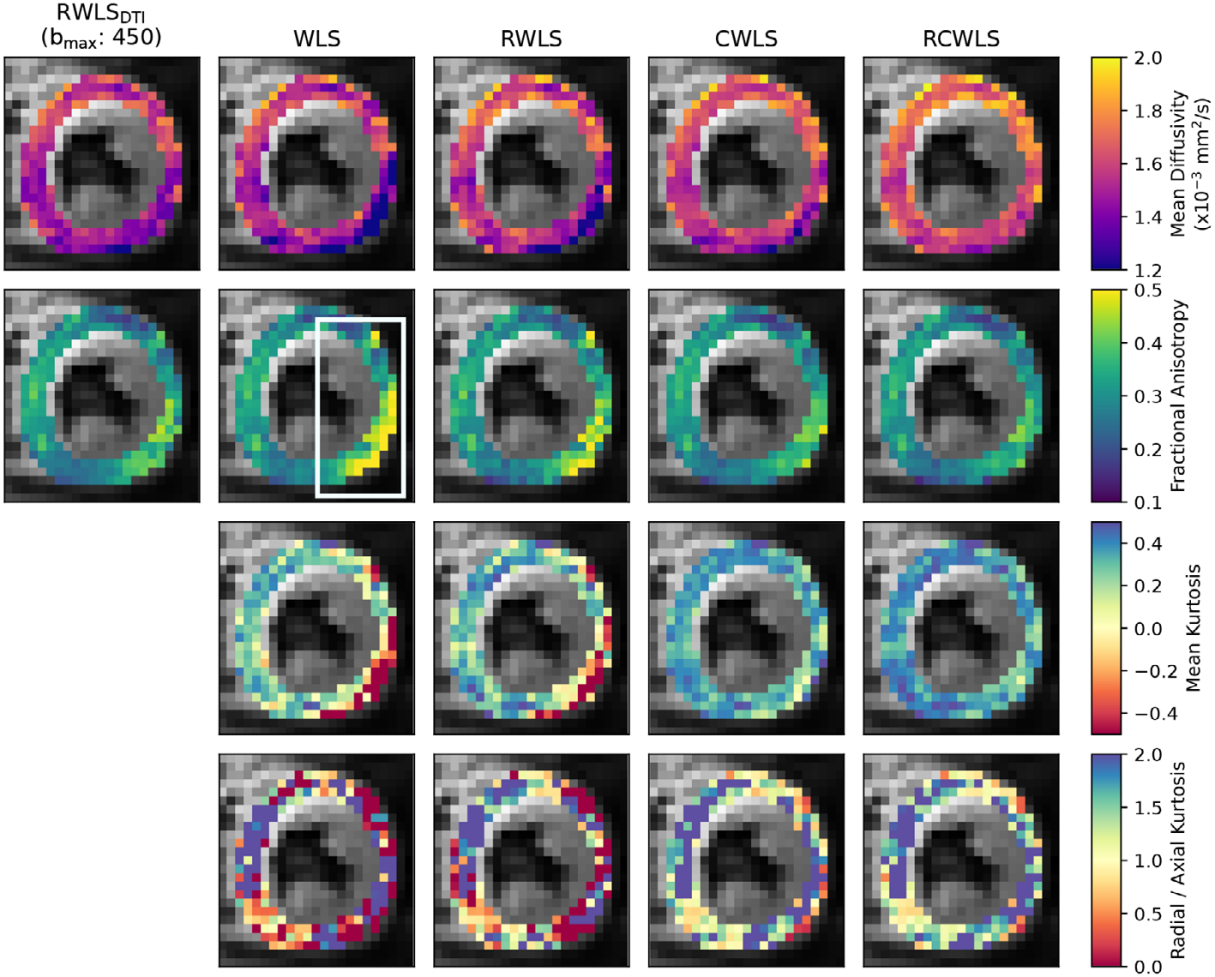
Measure maps for the mid slice of a single subject, for $b_{\max}=1350\;s/mm^{2}$. The first column shows MD and FA from DTI RWLS fitting ($b_{\max}=450\;s/mm^{2}$) for reference. All other columns show measures from DKI fits. Around 2 to 6 o'clock for the DKI fits is a region of implausibly low MD, high FA, and negative MK, for unconstrained fits. Constraints correct this region towards plausible values, with robust fitting visibly improving the region further in the same direction.

**FIGURE 7:**
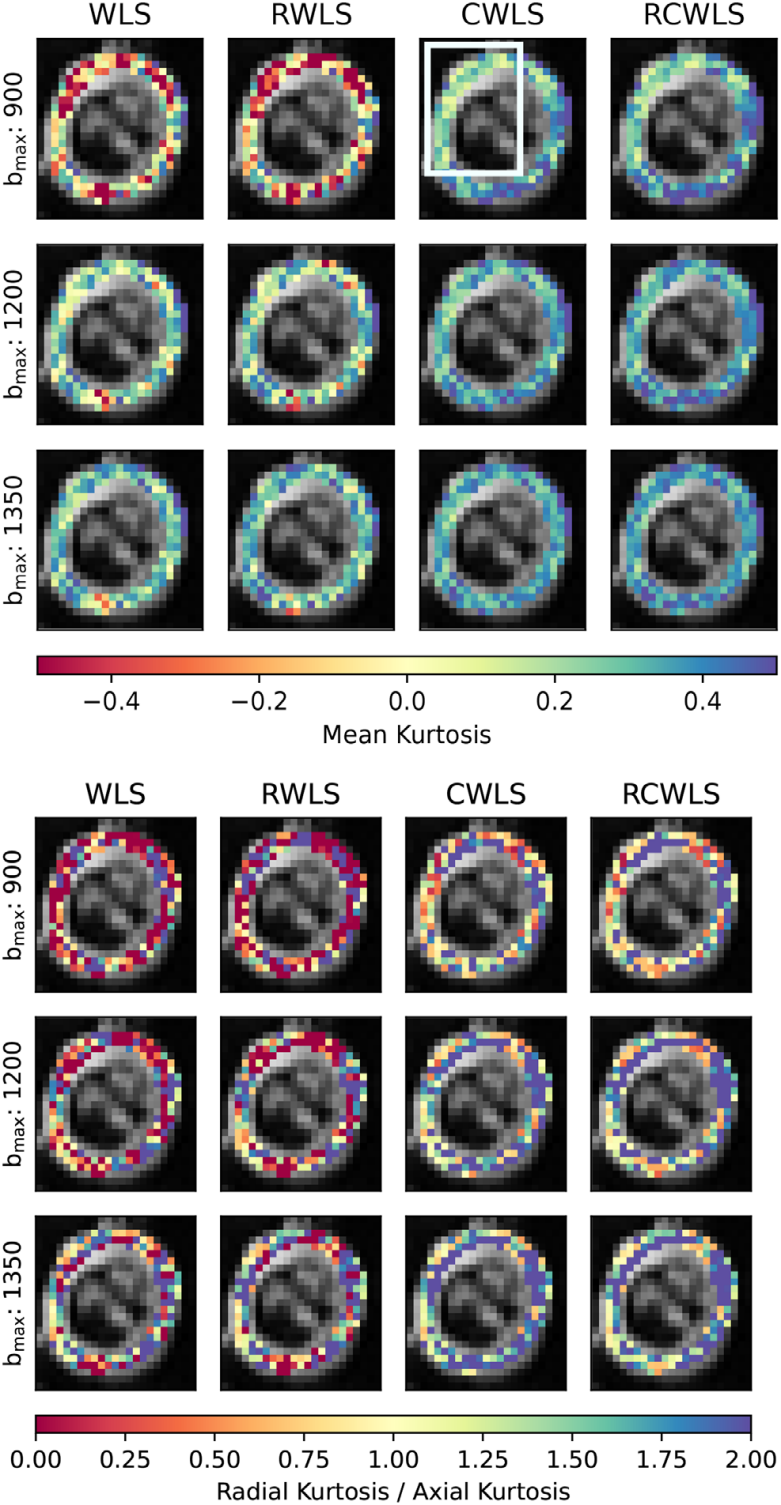
Mean kurtosis (MK) and radial kurtosis / axial kurtosis (RK/AK) maps for the basal slice of a single subject, for all $b_{\max}$ values (units $s/mm^{2}$) used for DKI fitting methods. Constraints increase MK given the same $b_{\max}$. For constrained fitting (CWLS and RCWLS), MK decreases with $b_{\max}$ in most voxels but increases between 8 and 12 o'clock. RCWLS for $b_{\max}=1350\;s/mm^{2}$ has the most spatially homogeneous MK.

**FIGURE 8:**
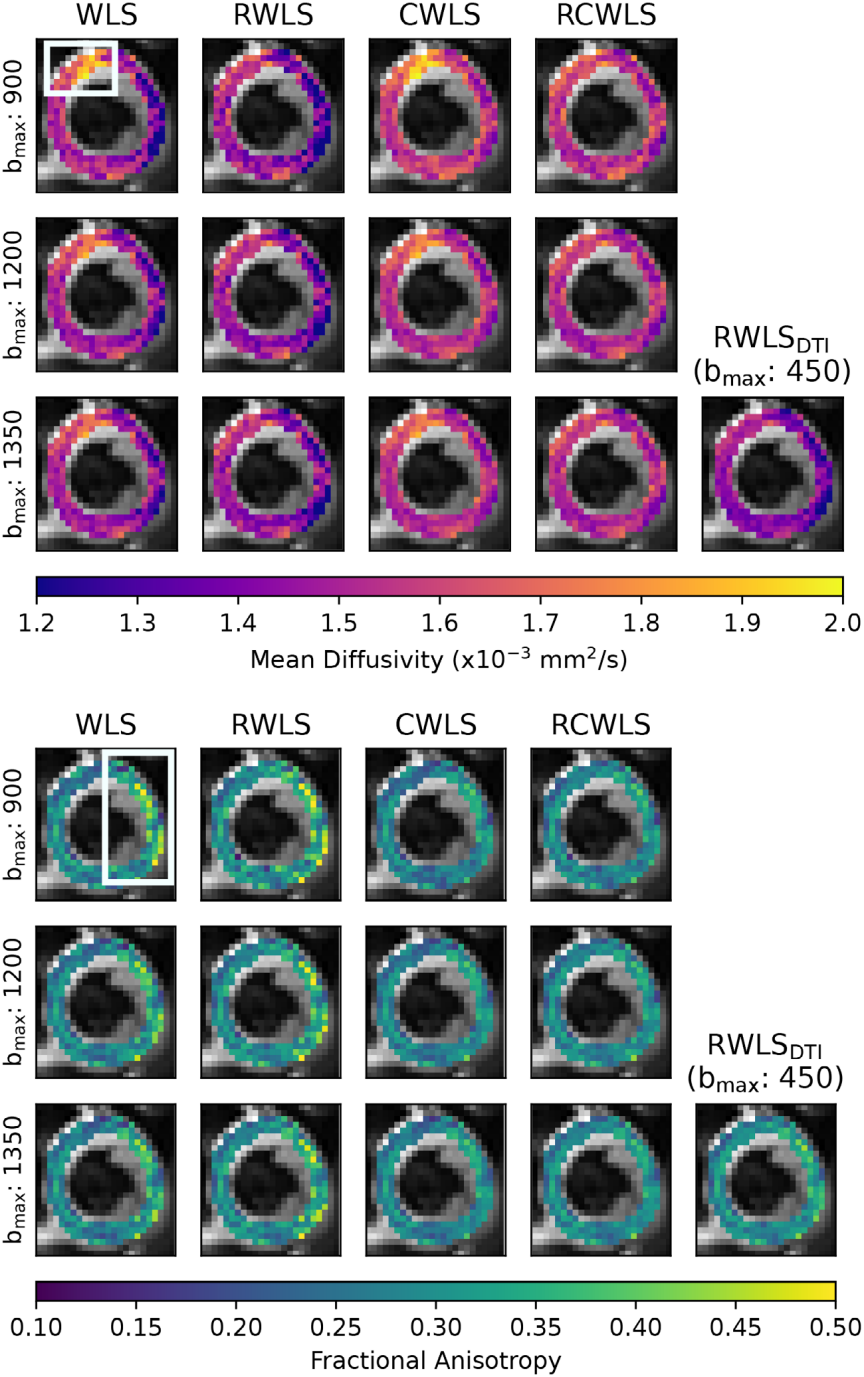
Mean diffusivity (MD) and fractional anisotropy (FA) maps for the basal slice of a single subject, for all $b_{\max}$ values (units $s/mm^{2}$) used for DKI fitting methods. Also shown are MD and FA from RWLS DTI fitting ($b_{\max}=450\;s/mm^{2}$) for reference. Robust fitting corrects elevated MD in the top‐left (10–12 o'clock), but constraints are needed to correct the reduced MD and elevated FA on the right (12–5 o'clock).

Figures 5, 6, 7, 8 show example measure maps for 4 different subjects. The colormap ranges were chosen based on the boxplots in Figure 1, in particular to emphasize whether values are above zero for MK or above one for RK/AK.

Figure 5 shows basal‐slice maps for $b_{\max}=1350\;s/mm^{2}$ for the subject with the second highest MD and MK for non‐robust fitting WLS/CWLS in Figure 1. Robust fitting gave large changes over the whole myocardium, bringing measures into better agreement with other subjects, such that for robust fitting methods this subject is no longer an outlier in the boxplots of Figure 1. MD and MK are reduced, and FA is increased, for robust methods vs non‐robust methods. Constraints increase MK and RK/AK, which is most noticeable between RWLS and RCWLS. In particular for RCWLS, RK/AK > 1 in nearly every voxel and is relatively homogeneous. This example shows that kurtosis can appear to be positive for non‐constrained methods, but these kurtosis measures can still be corrupted. Going from WLS to RWLS results in a large reduction of MK, indicating that its consistently positive value for WLS was likely due to corruptions in the data (which, in this case, also caused an inflation of MD). By adding constraints to robust fitting, that is, going from RWLS to RCWLS, consistently positive MK is recovered, but with much lower values than from the WLS fit.

Figure 6 shows mid‐slice maps for $b_{\max}=1350\;s/mm^{2}$ where robust fitting had large effects in a localized region (around 2 to 6 o'clock) with low MD, high FA, and negative MK for WLS. Here, both robust fitting and constraints independently increase MD, reduce FA, and increase MK, such that RCWLS gives the most spatially uniform values. The regions where RK/AK is around 1 for RCWLS coincide with relatively lower FA.

Figure 7 shows MK and RK/AK basal‐slice maps for different $b_{\max}$. Constraints give positive MK and make RK/AK > 1 overall. Reading across the rows, constraints increase MK and RK / AK given the same $b_{\max}$. Differences between unconstrained and constrained fitting decrease with $b_{\max}$. Reading down the RCWLS column, MK decreases with $b_{\max}$ for most voxels but increases between 8 and 12 o'clock. These opposing effects result in RCWLS for $b_{\max}=1350\;s/mm^{2}$ having the most homogeneous MK.

Figure 8 shows MD and FA maps in a basal slice for different $b_{\max}$ and for DTI fitting ($b_{\max}=450\;s/mm^{2}$). Robust fitting corrects elevated MD in the top‐left (10–12 o'clock), but constraints are needed to correct the reduced MD and elevated FA on the right (12–5 o'clock). The effects on MD and FA are consistent with the overall group trends. RCWLS gives the most homogeneous MD and FA, with the least variation with $b_{\max}$.

## 4 DISCUSSION

Our results show that both robust fitting and convexity constraints affect DTI/DKI measures in important ways. Figure 5 helps to show that the RCWLS results are not just the robust result with the negative kurtosis turned to zero—the kurtosis becomes convincingly positive, and all measures change when adding constraints. In this case, robust fitting reduced inflated MK caused by corruptions, while adding constraints increased MK. Figure 6 demonstrates a region where only robust fitting and constraints together appear to fully resolve a region of corrupted measures. In theory, RCWLS provides advantages greater than the sum of its parts: convexity constraints should make outlier identification easier, which should improve the weights in the final WLS fit.

### 4.1 Constraints

Imposing convexity constraints resulted in statistically significant differences in all measures for all $b_{\max}$ values. The precise changes, notably increased MK and RK/AK, are important. Only constrained fitting reliably shows RK > AK (for RCWLS $b_{\max}=1350\;s/mm^{2}$, RK/AK mean and median are 1.65 and 1.70 respectively), despite this being expected in myocardium since there are fewer restrictions to diffusion parallel to the long axes of cardiomyocytes.^3^, ^35^ It is worth emphasizing that adding constraints does not just turn negative kurtosis into zero kurtosis in certain voxels (something that could be trivially obtained in post‐processing the fitting results), but fundamentally alters the optimum coefficient vector (Figures 5 and 6).

### 4.2 Robustness

Robust fitting can have large overall and regional benefits on individual subjects, which is invaluable for potential clinical applications. Variation between subjects within a group ought to represent physiological variation (and non‐gross measurement error) rather than the effects of image corruptions. In the context of a study comparing different groups (e.g., healthy volunteers vs. disease), the reduction in the spread of measures from robust fitting can increase statistical power and lower the probability of a type 2 errors (false negatives).^21^

### 4.3 MD and FA

While it is known that MD and FA obtained from fitting the DKI signal representation can be different compared to those obtained from the DTI signal representation,^36^ constraints further modify these values: MD increases from DTI to DKI and from unconstrained to constrained fitting, but while FA increases from DTI to DKI, it decreases from unconstrained to constrained fitting, resulting in RCWLS giving the lowest FA values. This shows that the FA changes from DTI to DKI were largely in the context of parameter fits that violated constraints. To the best of our knowledge, other works noting differences in MD and FA between DTI and DKI have not utilized constraints or robust fitting.

### 4.4 $b_{\max}$ effect

Varying $b_{\max}$ allowed for investigating the importance of constraints and robustness for different experimental designs: higher $b_{\max}$ data is more informative about kurtosis but has lower SNR. While previous work has noted the dependence of the performance of (simplified models of) cardiac DKI on $b_{\max}$,^3^ it is noteworthy that MK and RK appear to increase with $b_{\max}$ for unconstrained fitting, but decrease with $b_{\max}$ for constrained fitting. This only serves to emphasize the importance of using fitting constraints. Due to these opposite trends, the differences between unconstrained and constrained fitting get smaller as $b_{\max}$ increases, suggesting fewer and milder constraint violations as data become more informative about kurtosis.

### 4.5 Limitations

Although the differences between non‐robust and robust methods were generally insignificant at the group level, the errors from non‐robust methods would make the detection of any actually existing differences quite challenging, which is perhaps ironic. It is also true that summary statistics such as mean measures over all voxels can be insensitive to the improvement in measure maps when using robust fitting, and yet post hoc statistical analysis of regions of interest (where measures were most changed by robust methods) would also seem problematic. The only measure that indicated any *potential* effect (at the group level) from robust fitting alone was RK/AK, particularly for $b_{\max}=1350\;s/mm^{2}$ (*p* = 0.024), but we cannot rule out a false positive here due to multiple comparisons of many measures. The ratio RK/AK might be more sensitive to changes from robust fitting, but more subjects would be required to have sufficient statistical power to determine this.

Higher $b_{\max}$ fits included all lower b‐values, and so there is more data available for these fits. Our study sought to determine how the results changed as more information about kurtosis was available, specifically in the context of understanding the effects of robust fitting and constrained fitting, so the conflation from having both higher b‐value data (which has lower SNR) and more data overall was not of particular concern. To perform an analysis about the suitability of different data designs, we believe many more subjects would be required, as well as a more nuanced analysis of trends with $b_{\max}$ (rather than just paired tests). This work could be restricted to robust and constrained fitting methods.

A limitation of signal representations, such as DTI and DKI, is a lack of clear insight into the specific quantitative links to the underlying biology: differences in kurtosis measures between groups can be observed and reasoned about, but the absolute values are not ascribed any particular meaning.^3^ Nonetheless, we can speculate that kurtosis measures (derived from appropriate fitting techniques) may be a way to increase the biomarker space and gain insight into disease; further work is required.

## 5 CONCLUSION

In this work, we have developed robust constrained weighted least squares (RCWLS), the first robust estimation technique for DKI that incorporates necessary constraints on the signal behavior. Using in vivo human cardiac DKI data from healthy volunteers collected with a Connectom scanner, we determined that RCWLS is the most suitable fitting technique compared with others that lack either robustness or constraints. For $b=1350\;s/mm^{2}$, RCWLS gave the following measures across subjects: MD $1.68\pm 0.050\;\times 10^{-3}{\text{mm}}^{2}/s$, FA 0.30±0.013, MK 0.36±0.027, AK 0.26±0.027, RK 0.42±0.040, and RK/AK 1.65±0.19. Constraints, but not robustness, had a significant effect on all diffusion and kurtosis measures. However, robust fitting corrected large errors for some subjects and generally improved diffusion and kurtosis maps. Only RCWLS convincingly showed radial kurtosis to be larger than axial kurtosis for all subjects, something that is expected in myocardium due to increased restrictions to diffusion in the plane perpendicular to the primary myocyte direction. RCWLS also showed the best correction of corrupted regions in diffusion parameter maps for individual subjects. Future work on in vivo cardiac DKI should utilize fitting techniques that are both robust and constrained, such as RCWLS.

## CONFLICT OF INTEREST

The authors declare no potential conflict of interests.

## APPENDIX A GRADIENT WAVEFORMS

Figure A1 shows the gradient waveforms used in this work. The timing of the diffusion encoding waveform is 17, 8, and 17 ms for the pre‐, pause‐, and post‐duration, respectively.

## APPENDIX B CONSTRAINT MATRICES

Many basis elements can be excluded a priori because they cannot contribute, leaving:

(B1)
$$\begin{array}{ll} e & =\left(s_{1},s_{2},s_{3},q_{1}s_{1},q_{1}s_{2},q_{1}s_{3},\right.\\ & \;{\left.q_{2}s_{1},q_{2}s_{2},q_{2}s_{3},q_{3}s_{1},q_{3}s_{2},q_{3}s_{3}\right)}^{T} \end{array}$$

We can parameterize the matrix H(θ) as

(B2)
$$2\left[\begin{array}{cccccccccccc} \theta_{1} & \theta_{2} & \theta_{4} & 0 & 0 & 0 & 0 & 0 & 0 & 0 & 0 & 0\\ \theta_{2} & \theta_{3} & \theta_{5} & 0 & 0 & 0 & 0 & 0 & 0 & 0 & 0 & 0\\ \theta_{4} & \theta_{5} & \theta_{6} & 0 & 0 & 0 & 0 & 0 & 0 & 0 & 0 & 0\\ 0 & 0 & 0 & \theta_{7} & \theta_{10} & \theta_{11} & \theta_{10} & \theta_{16} & \theta_{19} & \theta_{11} & \theta_{19} & \theta_{17}\\ 0 & 0 & 0 & \theta_{10} & \theta_{16} & \theta_{19} & \theta_{16} & \theta_{12} & \theta_{20} & \theta_{19} & \theta_{20} & \theta_{21}\\ 0 & 0 & 0 & \theta_{11} & \theta_{19} & \theta_{17} & \theta_{19} & \theta_{20} & \theta_{21} & \theta_{17} & \theta_{21} & \theta_{14}\\ 0 & 0 & 0 & \theta_{10} & \theta_{16} & \theta_{19} & \theta_{16} & \theta_{12} & \theta_{20} & \theta_{19} & \theta_{20} & \theta_{21}\\ 0 & 0 & 0 & \theta_{16} & \theta_{12} & \theta_{20} & \theta_{12} & \theta_{8} & \theta_{13} & \theta_{20} & \theta_{13} & \theta_{18}\\ 0 & 0 & 0 & \theta_{19} & \theta_{20} & \theta_{21} & \theta_{20} & \theta_{13} & \theta_{18} & \theta_{21} & \theta_{18} & \theta_{15}\\ 0 & 0 & 0 & \theta_{11} & \theta_{19} & \theta_{17} & \theta_{19} & \theta_{20} & \theta_{21} & \theta_{17} & \theta_{21} & \theta_{14}\\ 0 & 0 & 0 & \theta_{19} & \theta_{20} & \theta_{21} & \theta_{20} & \theta_{13} & \theta_{18} & \theta_{21} & \theta_{18} & \theta_{15}\\ 0 & 0 & 0 & \theta_{17} & \theta_{21} & \theta_{14} & \theta_{21} & \theta_{18} & \theta_{15} & \theta_{14} & \theta_{15} & \theta_{9} \end{array}\right]$$

The “slack matrix” L(α) is determined by Equation (7f). We can write $L(\alpha )=\sum_{u=1}\alpha_{u}L_{u}$, such that $e^{T}\cdot L_{u}\cdot e=0$. There are 18 such matrices, so we parameterize L(α) as

(B3)
$$\left[\begin{array}{cccccccccccc} 0 & 0 & 0 & 0 & \beta_{10} & \beta_{11} & 0 & \beta_{13} & \beta_{14} & 0 & \beta_{16} & \beta_{17}\\ 0 & 0 & 0 & \alpha_{10} & 0 & \beta_{12} & \alpha_{13} & 0 & \beta_{15} & \alpha_{16} & 0 & \beta_{18}\\ 0 & 0 & 0 & \alpha_{11} & \alpha_{12} & 0 & \alpha_{14} & \alpha_{15} & 0 & \alpha_{17} & \alpha_{18} & 0\\ 0 & \alpha_{10} & \alpha_{11} & 0 & 0 & 0 & 0 & \beta_{1} & \beta_{2} & 0 & \beta_{4} & \beta_{5}\\ \beta_{10} & 0 & \alpha_{12} & 0 & 0 & 0 & \alpha_{1} & 0 & \beta_{3} & \alpha_{4} & 0 & \beta_{6}\\ \beta_{11} & \beta_{12} & 0 & 0 & 0 & 0 & \alpha_{2} & \alpha_{3} & 0 & \alpha_{5} & \alpha_{6} & 0\\ 0 & \alpha_{13} & \alpha_{14} & 0 & \alpha_{1} & \alpha_{2} & 0 & 0 & 0 & 0 & \beta_{7} & \beta_{8}\\ \beta_{13} & 0 & \alpha_{15} & \beta_{1} & 0 & \alpha_{3} & 0 & 0 & 0 & \alpha_{7} & 0 & \beta_{9}\\ \beta_{14} & \beta_{15} & 0 & \beta_{2} & \beta_{3} & 0 & 0 & 0 & 0 & \alpha_{8} & \alpha_{9} & 0\\ 0 & \alpha_{16} & \alpha_{17} & 0 & \alpha_{4} & \alpha_{5} & 0 & \alpha_{7} & \alpha_{8} & 0 & 0 & 0\\ \beta_{16} & 0 & \alpha_{18} & \beta_{4} & 0 & \alpha_{6} & \beta_{7} & 0 & \alpha_{9} & 0 & 0 & 0\\ \beta_{17} & \beta_{18} & 0 & \beta_{5} & \beta_{6} & 0 & \beta_{8} & \beta_{9} & 0 & 0 & 0 & 0 \end{array}\right]$$

where $\beta_{ij}=-\alpha_{ij}$ (where $\beta_{ij}$ are only used here to assist with display, they are not additional parameters).

The reason for writing the sum of squares representation as matrix H(θ)+L(α) in Equation (7b), and therefore introducing parameters α, can be understood by considering (7e): there are (infinitely) many choices for H(θ) given basis Equation (B1), but we must implement a specific matrix H(θ) (e.g., Equation B2). The flexibility is then explicitly parameterized via L(α).

## APPENDIX C REDUCED CONSTRAINED PROBLEM

As explained in the appendix of,^19^ the size of the semidefinite programming problem can be reduced (giving significant computational savings) by replacing Equation (7a) with

(C1)
$$\begin{array}{l} {arg\;min}_{\theta ,\alpha }||{V^{\prime }}^{T}\cdot \theta -{V^{\prime }}^{T}\cdot {\hat{\theta }}_{\text{WLS}}|{|}^{2} \end{array}$$

where ${\hat{\theta }}_{\text{WLS}}$ is the unconstrained WLS solution given by Equation (5) and V is given by the Cholesky factorization $\left({X^{\prime }}^{T}\cdot X^{\prime }\right)=V\cdot V^{T}$. For RCWLS the problem is reduced at each iteration (the *unconstrained* fit result ${\hat{\theta }}_{\text{WLS}}$ depends on the previous *constrained* fit). The semi‐definite program Equation (7) only needs solving if the WLS estimate violates the constraints.

## References

[1] Alexander DC , Barker GJ , Arridge SR . Detection and modeling of non‐Gaussian apparent diffusion coefficient profiles in human brain data. Magn Reson Med. 2002;48:331‐340. 12210942 10.1002/mrm.10209

[2] Jensen JH , Helpern JA , Ramani A , Lu H , Kaczynski K . Diffusional kurtosis imaging: the quantification of non‐gaussian water diffusion by means of magnetic resonance imaging. Magn Reson Med. 2005;53:1432‐1440. 15906300 10.1002/mrm.20508

[3] McClymont D , Teh I , Carruth E , et al. Evaluation of non‐Gaussian diffusion in cardiac MRI. Magn Reson Med. 2017;78:1174‐1186. 27670633 10.1002/mrm.26466 PMC5366286

[4] Jensen JH , Helpern JA . MRI quantification of non‐Gaussian water diffusion by kurtosis analysis. NMR Biomed. 2010;23:698‐710. 20632416 10.1002/nbm.1518 PMC2997680

[5] Tabesh Ali A , Jensen JH , Ardekani BA , Helpern JA . Estimation of tensors and tensor‐derived measures in diffusional kurtosis imaging. Magn Reson Med. 2011;65:823‐836. 21337412 10.1002/mrm.22655 PMC3042509

[6] Basser PJ , Pierpaoli C . Microstructural and physiological features of tissues elucidated by quantitative‐diffusion‐tensor MRI. J Magn Reson. 2011;213:560‐570. 22152371 10.1016/j.jmr.2011.09.022

[7] Russell GG , Helpern JA , Tabesh A , Jensen JH . Quantitative assessment of diffusional kurtosis anisotropy. NMR Biomed. 2015;28:448‐459. 25728763 10.1002/nbm.3271 PMC4378654

[8] Hansen B , Shemesh N , Jespersen SN . Fast imaging of mean, axial and radial diffusion kurtosis. Neuroimage. 2016;142:381‐393. 27539807 10.1016/j.neuroimage.2016.08.022 PMC5159238

[9] Hansen B , Lund TE , Sangill R , Jespersen SN . Experimentally and computationally fast method for estimation of a mean kurtosis. Magn Reson Med. 2013;69:1754‐1760. 23589312 10.1002/mrm.24743

[10] Jespersen SN . White matter biomarkers from diffusion MRI. J Magn Reson. 2018;291:127‐140. 29705041 10.1016/j.jmr.2018.03.001

[11] Henriques Rafael N , Correia MM , Nunes RG , Ferreira HA . Exploring the 3D geometry of the diffusion kurtosis tensor—impact on the development of robust tractography procedures and novel biomarkers. Neuroimage. 2015;111:85‐99. 25676915 10.1016/j.neuroimage.2015.02.004

[12] Henriques RN , Jespersen SN , Jones DK , Veraart J . Toward more robust and reproducible diffusion kurtosis imaging. Magn Reson Med. 2021;86:1600‐1613. 33829542 10.1002/mrm.28730 PMC8199974

[13] Teh I , David S , Boyle JH , et al. Cardiac q‐space trajectory imaging by motion‐compensated tensor‐valued diffusion encoding in human heart in vivo. Magn Reson Med. 2023;90:150‐165. 36941736 10.1002/mrm.29637 PMC10952623

[14] Hanson CA , Kamath A , Gottbrecht M , Ibrahim S , Salerno M . T2 relaxation times at cardiac MRI in healthy adults: a systematic review and meta‐analysis. Radiology. 2020;297:344‐351. 32840469 10.1148/radiol.2020200989 PMC7605362

[15] Afzali M , Mueller L , Coveney S , et al. In vivo diffusion MRI of the human heart using a 300 mT/m gradient system. Magn Reson Med. 2024;92:1022‐1034. 38650395 10.1002/mrm.30118 PMC7617480

[16] Afzali M , Mueller L , Coveney S , et al. Quantification of non‐Gaussian diffusion in the human heart in vivo. Proceedings of the Annual Meeting of ISMRM. 2024.

[17] Afzali M , Coveney S , Mueller L , et al. Cardiac diffusion kurtosis imaging in the human heart in vivo using 300 mT/m gradients. Magn Reson Med. 2025;94:2100‐2112. doi:10.1002/mrm.30626 40605816 10.1002/mrm.30626 PMC12393209

[18] Tax CMW , Otte WM , Viergever MA , Dijkhuizen RM , Leemans A . REKINDLE: robust extraction of kurtosis INDices with linear estimation: Rekindle. Magn Reson Med. 2015;73:794‐808. 24687400 10.1002/mrm.25165

[19] Tom DH , Evren Ö , Aasa F . Enforcing necessary non‐negativity constraints for common diffusion MRI models using sum of squares programming. Neuroimage. 2020;209:116405. 31846758 10.1016/j.neuroimage.2019.116405

[20] Coveney S , Kelly C , Teh I , et al. Semi‐automated rejection of corrupted images in cardiac diffusion tensor imaging. Proceedings of the Annual Meeting of ISMRM. 2023.

[21] Coveney S , Afzali M , Mueller L , et al. Outlier detection in cardiac diffusion tensor imaging: shot rejection or robust fitting? Med Image Anal. 2025;101:103386.39667253 10.1016/j.media.2024.103386

[22] Veraart J , Van Hecke W , Sijbers J . Constrained maximum likelihood estimation of the diffusion kurtosis tensor using a Rician noise model. Magn Reson Med. 2011;66:678‐686. 21416503 10.1002/mrm.22835

[23] Magnani A , Lall S , Boyd S . Tractable Fitting with Convex Polynomials Via Sum‐of‐Squares. In: Proceedings of the 44th IEEE Conference on Decision and Control. IEEE; 2005:1672‐1677.

[24] Salvador R , Peña A , Menon DK , Carpenter TA , Pickard JD , Bullmore ET . Formal characterization and extension of the linearized diffusion tensor model. Hum Brain Mapp. 2005;24:144‐155. 15468122 10.1002/hbm.20076 PMC6871750

[25] Collier Q , Veraart J , Jeurissen B , Dekker AJ , Sijbers J . Iterative reweighted linear least squares for accurate, fast, and robust estimation of diffusion magnetic resonance parameters: IRLLS for estimation of diffusion MR parameters. Magn Reson Med. 2015;73:2174‐2184. 24986440 10.1002/mrm.25351

[26] Eleftherios G , Matthew B , Bagrat A , et al. Dipy, a library for the analysis of diffusion MRI data. Front Neuroinform. 2014;8(8). 10.3389/fninf.2014.00008 PMC3931231 24600385

[27] Sjölund J , Szczepankiewicz F , Nilsson M , Topgaard D , Westin C‐F , Knutsson H . Constrained optimization of gradient waveforms for generalized diffusion encoding. J Magn Reson. 2015;261:157‐168. 26583528 10.1016/j.jmr.2015.10.012 PMC4752208

[28] Szczepankiewicz F , Sjölund J , Dall'Armellina E , et al. Motion‐compensated gradient waveforms for tensor‐valued diffusion encoding by constrained numerical optimization. Magn Reson Med. 2021;85:2117‐2126. 33048401 10.1002/mrm.28551 PMC7821235

[29] Mueller L , Afzali M , Coveney S , et al. ZOOM and enhance: ZOnally magnified oblique multi‐slice for cardiac DTI with ultra‐strong gradients. Proceedings of the Annual Meeting of ISMRM. 2024.

[30] Jens S . *jsjol/NOW*. original‐date: 2017–03‐01T09:06:49Z. 2024.

[31] Symms MR , Wheeler‐Kingshott CA , Parker GJM , Barker GJ . ZOnally‐magnified oblique multislice (ZOOM) EPI. In: Proceedings of the International Society for Magnetic Resonance in Medicine (ISMRM). Vol 160. ISMRM; 2000.

[32] Lauenstein TC , Sharma P , Hughes T , Heberlein K , Tudorascu D , Martin DR . Evaluation of optimized inversion‐recovery fat‐suppression techniques for T2‐weighted abdominal MR imaging. J Magn Reson Imaging. 2008;27:1448‐1454. 18504735 10.1002/jmri.21350

[33] Lowekamp BC , Chen DT , Ibáñez L , Blezek D . The design of SimpleITK. Front Neuroinform. 2013;7:45. 24416015 10.3389/fninf.2013.00045 PMC3874546

[34] Ben‐Amitay S , Jones DK , Assaf Y . Motion correction and registration of high b‐value diffusion weighted images. Magn Reson Med. 2012;67:1694‐1702. 22183784 10.1002/mrm.23186

[35] Tracy RE , Sander GE . Histologically measured cardiomyocyte hypertrophy correlates with body height as strongly as with body mass index. Cardiol Res Pract. 2011;2011:658958. 21738859 10.4061/2011/658958 PMC3123935

[36] Lu H , Jensen JH , Ramani A , Helpern JA . Three‐dimensional characterization of non‐gaussian water diffusion in humans using diffusion kurtosis imaging. NMR Biomed. 2006;19:236‐247. 16521095 10.1002/nbm.1020

